# A prospective controlled study: Minimally invasive stereotactic puncture therapy versus conventional craniotomy in the treatment of acute intracerebral hemorrhage

**DOI:** 10.1186/1471-2377-11-76

**Published:** 2011-06-23

**Authors:** Houguang Zhou, Yu Zhang, Ling Liu, Xu Han, Yinghong Tao, Yuping Tang, Wei Hua, Jianzhong Xue, Qiang Dong

**Affiliations:** 1 Department of Geriatrics, Huashan Hospital, Fudan University, Shanghai, China; 2 Department of Neurology, Jinling Hospital, Nanjing University Medicine School, Nanjing, China; 3 Department of Neurology, Huashan Hospital, Fudan University, Shanghai, China; 4 Department of General Medicine, Ouyang Hospital, Hongkou District, Shanghai, China; 5 Department of Neurosurgery, Huashan Hospital, Fudan University, Shanghai, China; 6 Department of Neurology, Affiliated ChangShu Hospital, Yangzhou University, Changshu, China

## Abstract

**Background:**

Spontaneous intracerebral hemorrhage (ICH) is a devastating form of stroke with the high mortality twofold to sixfold higher than that for ischemic stroke. But the treatment of haematomas within the basal ganglia continues to be a matter of debate among neurologists and neurosurgeons. The purpose of this study is to judge the clinical value of minimally invasive stereotactic puncture therapy (MISPT) on acute ICH.

**Methods:**

A prospective controlled study was undertaken. The clinical trial was in compliance with the WMA Declaration of Helsinki - Ethical Principles for Medical Research Involving Human Subjects. According to the enrollment criterion, there were 168 acute ICH cases analyzed, of which 90 cases were performed by MISPT ( MISPT group, MG) and 78 cases by Conventional craniotomy (CC group, CG), by means of compare of Glasgow Coma Scale(GCS) score, postoperative complications(PC) and rebleeding incidence(RI), moreover, long-term outcome of 1 year postoperation judged by Glasgow Outcome Scale (GOS), Barthel Index (BI), modified Rankin Scale (mRS) and case fatality(CF).

**Results:**

MG patients showed obvious amelioration in GCS score compared with that of CG. The total incidence of PC in MG decreased obviously compared with that of CG. The incidences of rebleeding in MG and CG were 10.0% and 15.4% respectively. There was no obvious difference between CFs of MG and CG. For three parameters representing long-term outcome, the GOS, BI and mRS in MG were ameliorated significantly than that of CG.

**Conclusion:**

These data suggested that the advantage of MISPT was displayed in minute trauma and safety, and seemed to be feasible and to had a trend towards improved long-term outcome.

**Trial Registration:**

The Australian New Zealand Clinical Trials Registry (ANZCTR), the registration number:ACTRN12610000945022.

## Background

Spontaneous intracerebral hemorrhage (ICH) comprises in 8-30% of all stroke victims, depending on regional and ethnic differences, and is a devastating form of stroke with the high mortality twofold to sixfold higher than that for ischemic stroke [1], and 1-year survival rate less than 50%[2]. Hitherto, morbidity and mortality following ICH remain the highest among all forms of cerebrovascular diseases, with a 30-day mortality rate of 35% to 52%, half of the deaths occurring in the first 2 days [3-5]. After initial irreversible tissue injury is suffered near the hemorrhage nidus, a progressive cascade of elevated local pressures, edema, and excitotoxicity causes additional secondary injury to surrounding brain [6-8]. Secondary brain injury by hematoma often occurs in the days following the initial hemorrhage and is intimately associated with significant neurological deterioration.

Despite being considerably frequent, the treatment of haematomas within the basal ganglia continues to be a matter of debate among neurologists and neurosurgeons. Present powerful evidence provided by the results of the International Study of the Treatment of Intracranial Hemorrhage (STICH) corroborates this statement which has been established before: there was not significant benefit for conventional aggressive surgical treatment over conservative medical treatment for the acute care of ICH [9]. Nevertheless, more than 7000 patients with ICH in the United States ever undergo traditional evacuation procedures each year [10].

However, various clinical studies testing the hypothesis that clot burden plays a significant role in several forms of intracranial hemorrhage have become available in recent years which seem to suggest that clot reduction plays an important role in limiting brain edema and additional neuronal injury, as well as in reducing the severity of neurological deficits following ICH [11-14]. Because of being attributed to the lack of validated therapeutic options for this form of stroke, the role of minimally invasive surgery (MIS) in the treatment of ICH has gained importance, and emerged several differrent operation methods over the past decade. In this context, our treatment with a stereotactic technique, which we have termed the minimally invasive stereotactic puncture therapy (MISPT), is herewith presented.

MISPT is a novel operative technique for ICH, which is developed by Pro Jia of China in 1997. Although several clinical studies on MISPT in acute phase of ICH are well recognized in the past decade, the impact of MISPT in short-term and long-term on neurological function of patients who survive the acute phase is less clear. The purpose of the present study was to investigate whether MISPT could maintain long-term benefit as short-term benefit and whether this method could improve ultimate outcomes in these ICH patients. Therefore we compared the long-term outcome one year after treatment obtained in a consecutive series of ICH patients treated by MISPT with the results achieved in a comparable group of patients who were treated by conventional craniotomy (CC).

## Methods

A prospective controlled study was undertaken. All ICH patients came from in-hospital from 2005 to 2008, diagnosed as ICH according to the ICH criteria of which is drafted by ASA [15]. The clinical trial was in compliance with the WMA Declaration of Helsinki - Ethical Principles for Medical Research Involving Human Subjects, and was performed with the approval of our hospital ethics committee(Reference number: JSCS2005058). We generated the sequence for enrolling a subject and allocating the treatment by a randomized number generated by computer. All cases have been monitored in a dedicated stroke unit. Volume of the ICH in milliliters was estimated on the basis of approximate ellipse volume with the A×B×C/2 formula, where A represents the largest diameter of the hematoma on axial CT cuts in centimeters, B the diameter of hematoma perpendicular to A on the same cut, and C the number of CT slices in which hematoma is visible multiplied by the slice thickness in centimeters [16,17].

### Inclusion and exclusion criteria for patients

#### Inclusion criteria were as follows

(1) diagnosed as having spontaneous hemorrhage in the basal ganglion or brain lobe of the brain by CT scan; (2) hemorrhage volume: 30-100 ml; (3) age range: 40-75 years; (4) muscle strength of the paralyzed limbs: grades 0-3 on the muscle strength scale; (5) hemorrhagic duration (from stroke onset to hospital) within 24 h; (6) informed consent from patients and/or their law representative.

#### Exclusion criteria were as follows

(1) disturbances of blood coagulation, such as thrombocytopenia, hepatitis, etc; (2) traumatic intracranial hemorrhage; (3)intracranial or general infection; (4) complicated with serious heart, liver, renal or lung disease or functional failure; (5) a previous stroke history with neurological deficits; (6) intracranial aneurysm or arteriovenous malformation complicated with hemorrhage; (7) consent form cannot be obtained from the patient herself or her law representative.

### Treatment methods

#### Minimally invasive stereotactic puncture and thrombolysis therapy (MISPT)

**Figure 1:**
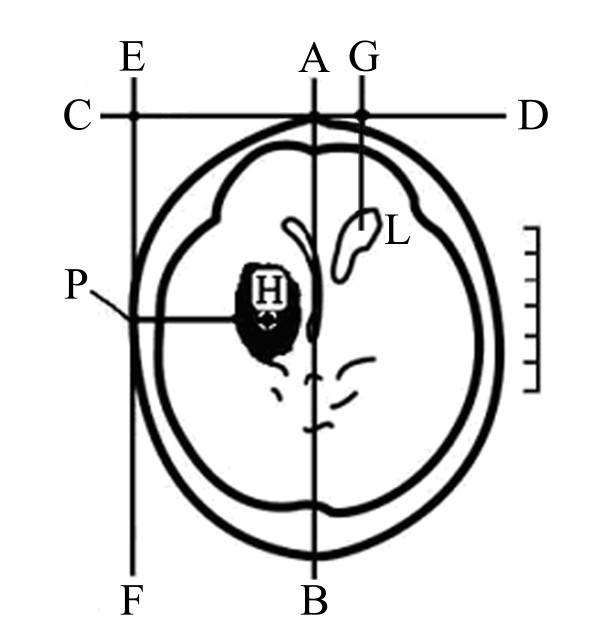
**The slice of the largest hematoma area and puncture point in the CT are illustrated**. AB median sagittal line, CD precoronal line, EF lateral sagittal line, P puncture point of hematoma, H center of hematoma, G puncture point of lateral cerebral ventricle, PH puncture depth, L lateral cerebral ventricle

All operations were performed under local anesthesia and intravenous sedation unless the patient was already intubated for medical or neurological indications independent of the procedure. Stereotactic aspiration of the haematomas was performed in the acute phase between the 6th and the 24th hour after onset of stroke refer to MISPT guide. Firstly, the target points was defined according to the computer tomography and mostly target points were chosen in the scan with the largest expansion of the haematoma(shown in Figure 1). Puncture situs was measured and marked on head noticing to get out of the way of main blood vessel, then puncture needle of suitable length(Type YL-1) was fixed on the operative electric drill. The puncture needle was perforated into predetermined depth, then the probe core removed, hematoma drawn out gently by syringe (diluted by saline solution if blood thicken) until 1/3 of hematoma were removed, needle-like hematoma disintegrator inserted. When no more blood could be syringed, the haematoma cavity was thoroughly rinsed with saline, until the saline fluid could be re-aspirated clearly.

To confirm gross haematoma evacuation, an immediate postoperative CT scan was taken for assessment of puncture needle placement and residual hematoma volume. If the placement of puncture needle is in the center of hematoma and stable, without rebleeding, the drainage-bag maintained 10 cm upon the head was linked to the puncture needle and switched for drainage. Once rebleeding, 1ku reptilase or 1 mg adrenalin should be injected into hematoma, drained after 0.5 h, and rinsed after 6-8 h. All patients were administered in a dedicated intensive care unit, where subsequent thrombolysis and clot drainage were performed at the bedside using sterile technique. Hematoma was continuiosly liquefied by liquefacient (containing 20000U-40000U urokinase/2-3 mL saline solution) for 2-4 days (3-5 times per day). In the 1st,3rd,5th and 7th day of postoperation, patients were re-examined the computer tomography. For haematoma difficulty to aspirate, it should be liquefied as far as possible at first and repeatedly aspirated using agitation manoeuvre of liquefacient to form performance vacuity. Haematoma breaking into one lateral cerebral ventricle should be perforated only haematoma cavity, but for those of haematoma breaking into both lateral cerebral ventricle and casting mould, haematoma cavity and opposite side lateral cerebral ventricle should be perforated simultaneously.

In addition, lumbar puncture could be performed to repeatedly replace cerebral spinal fluid using NS until color pale for 4-5 days (one time per day). When the hematoma was basically cleared or the reminder volume were less than 10 ml, the general condition of patients were stable, and intracranial pressure were normal by lumbar puncture, the puncture needle could be pull out if without intracranial hypertension after drainage tube occluded for 24 h. The puncture needle was removed at the bedside under sterile technique, and a single suture was placed at its exit site and covered with an occlusive dressing. Additionally, all patients received routine medical treatment.

#### Conventional craniotomy(CC)

After 6-24 h of onset, clearance of hematoma by traditional craniotomy with large bone flap removed was operated in Department of Neurosurgery. The surgery was assessed by postoperative CT to determine if it was successful or not. Meantime, the routine medical treatment was performed.

#### Complications observation

To comparing its therapeutic effect, several main clinical complications including pulmonary infection, digestive tract hemorrhage and epilepsy were observed two week postoperation. Meanwhile, incidence of complication and rebleeding observation were also carried out.

### Follow-Up and Outcome Assessment

Evaluation of all patients followed identical criteria. Initial assessment included baseline characteristics (age, gender, haematoma volume, et al). The pretreatment clinical state of this cohort was assessed according to the GCS. All cases between two groups could be matched each other with regard to baseline characteristics. The posttreatment clinical state of patients was assessed according to the GSC score, incidence of complication, recurrence of bleeding after surgery. Outcome 1 year after stroke was the major endpoint. Four outcome parameters commonly used to assess outcome were employed to study the full impact of haemorrhagic stroke on long-term follow-up. Total case fatality was defined 1 year after stroke. According to the Glasgow Outcome Scale (GOS), clinical outcomes were graded, ranging from good recovery (GOS 5) to dead (GOS 1). The performance and functional status of the patients in activities of daily living (ADL) were measured with the Barthel Index (BI). Handicap was assessed using the modified Rankin Scale (mRS). The datas of all four outcome parameters for two treatment groups were analysed and compared.

### Statistical Analysis

Categorical variables were analysed using *χ*^2 ^test or Fisher's exact test for small numbers. Measurement data were analysed using T-tests. All recorded data were input using Epi Info software and statistically analysed using SPSS 11.5 statistical software. For all analyses, p < 0.05 was considered to be statistically significant.

## Results

There were 168 patients analyzed, of which 90 cases were treated with MISPT and 78 cases with CC. There were no statistically significant difference in sex, age, GCS score, localization of bleeding, hemorrhage volume, the level of blood pressure while hospitalization and duration of hypertension in the three groups (shown in Table 1).

**Table 1:** The Baseline Characteristics of Patients

Group	MISPT patients	CC patients	P value
Number of patients	90	78	
Gender (m:f)	59:31	50:28	0.844
Mean age (years)	57.6 ± 11.2	59.2 ± 10.7	0.341
GCS score (n/%)			0.885
4-5	7/7.8	8/10.3	
6-9	38/42.2	35/44.9	
10-12	27/30.0	20/25.6	
13-15	18/20.0	15/19.2	
Mean haematoma volume (n/%)			0.856
30-59(mL)	33/36.7	26/33.3	
60-79(mL)	30/33.3	29/37.2	
80-100(mL)	27/30.0	23/29.5	
Direction of the haematoma (n/%)			0.550
Left-sided	48/53.3	38/51.3	
Right-sided	42/46.7	40/48.7	
Location of the haematoma (n/%)			0.571
Basal ganglia	48/53.3	39/50.0	
Deep brain lobe	26/28.9	20/25.6	
Thalamus	16/17.8	19/24.4	
Mean BP			
SBP	174.5 ± 13.2	172.9 ± 11.5	0.414
DBP	102.2 ± 8.3	99.2 ± 7.6	0.361
Duration of BP (years)	6.7 ± 2.0	7.1 ± 2.4	0.219

### Comparison of consciousness level after surgery, GCS score and the incidence of complications between the two groups

**Figure 2:**
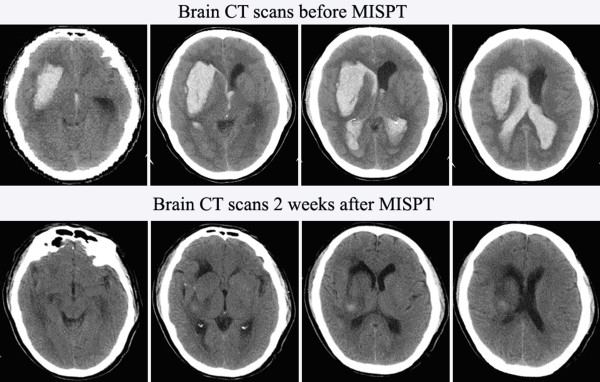
**CT scans of one patient in a coma(GCS score 6)with a huge haematoma(> 70 mL) before MISPT(upper), and CT scans of the same patient in consciousness (GCS score 14) 2 weeks after completed MISPT with urokinase (lower)**. All scans demonstrate a series size of hematoma on axial imaging.

The GCS score had no significant difference between two groups before operation(8.1 ± 2.3 and 8.4 ± 3.2 respectively, P = 0.523). The total incidence of complication in the MISPT group was lower than that of the CC group (32.3% and 80.7% respectively, P = 0.001). Pulmonary infection(8.9%), digestive tract hemorrhage(17.8%) and epilepsy(5.6%) in the MISPT group were all lower than that of the CC group (21.8%, 39.7% and 19.2%, respectively) (P = 0.029, P = 0.002 and P = 0.036, respectively). The incidence of bleeding recurrence had no significant difference between two groups (10.0% and 15.4% respectively, P = 0.051) (shown in Table 2). According to post-operative imaging findings, about 70-80% of haematoma could be removed in the MISPT group. There were some typical examples in this study. For example, CT scans of one coma patient (GCS score 6)with a huge haematoma (> 70 mL) were shown(Figure 2). It was clear that there were obvious difference in haematoma volume and edema of surrounding brain before MISPT with consciousness of GCS score 5 and 2 weeks after MISPT with consciousness of GCS score 14. The patient achieved self-care living 1 year after on-set(shown in Figure 2).

**Table 2:** Consciousness Level after Surgery, GCS Score and Incidence of Complication of Two Groups

Group	MISPT patients	CC patients	P value
Number of patients GCS score	90	78	
before operation	8.1 ± 2.3	8.4 ± 3.2	0.523
after operation	10.5 ± 0.9	8.0 ± 0.6	0.039
incidence of complication(n/%)	29/32.3	63/80.7	0.000
pulmonary infection	8/8.9	17/21.8	0.019
digestive tract hemorrhage	16/17.8	31/39.7	0.002
epilepsy	5/5.6	15/19.2	0.006
Bleeding recurrence (n/%)	9/10.0	12/15.4	0.293

### Comparison of long-term outcome and total case fatality of two groups 1 year after stroke

Although there was no sharp difference in case fatality between MISPT group and CC group 1 year after stroke(18.9% and 24.4% respectively, P = 0.096), the results showed that the recovery of survival in MISPT group was significantly better than that of the CC group according to the assession results of GOS, mRS and BI(GOS, P = 0.000; mRS, P = 0.001; BI, P = 0.000, respectively). (shown in Table 3).

**Table 3:** Outcome 1 Year after Stroke of Two Groups

Group	MISPT patients	CC patients	P value
Number of patients	90	78	
case fatality(n/%)	17/18.9	19/24.4	0.389
GOS	4.3 ± 0.4	2.6 ± 0.3	0.043
mRS	2.2 ± 0.3	3.9 ± 0.4	0.042
BI	79.5 ± 11.1	62.0 ± 9.8	0.011

## Discussion

The mass effect of hematoma can lead to the brain damage such as intracranial hypertension or cerebral hernia [18]. But there were some evidences shown that the mass effect caused by hematoma volume(< 60 ml) was not the dominant injury mechanism, whereas the toxic substances released from the hematoma were the most important factor in the pathological mechanism of the cerebral hemorrhage [19-21]. It was reported that elevated levels of glutamate were found in the perihematomal region after ICH and were decreased during hematoma drainage. Conversely, ischemic LPRs were not found in perihematomal regions and were unchanged during hematoma drainage. These data suggest that excitotoxicity related to glutamate may have an important impact on secondary injury. The data failed to support the role of ischemia in secondary perihematomal damage [6].

Therefore, effective removal of hematoma at the acute phase is the crucial principle of treatment with ICH for saving life and improving the long-term quality of life. CC by removing bone flap is the classical technique treating ICH which is characterized by good view, clearance of hematoma completely, easy haemostasia, and the entire reduction of pressure, but also have some shortcomings such as taking long time to operate, damaging brain severely, being subject to pulling the brain in the operation, the brain tissue around the hematoma readily damaged by electrocoagulation, re-bleeding readily, producing a series of pathophysiological changes in post-operation (such as disturbance of water and electrolyte, fluctuation of blood sugar, instability of life signs and so on), which resulting in severe impairment of neurological function, multiple complications, higher invalidism rate and fatality rate. With regard to basal ganglia haemorrhages, comparing the outcome of patients treated surgically with that of patients managed conservatively, many earlier and current publications showed no benefit from conventional surgery [9,10,22-25]. Correspondly this disappointing results, only a few reports have demonstrated a trend towards better outcomes in conventional surgical group [26-28]. While analysing the given data, it becomes obvious that the major problem in all these studies is the heterogeneity of the ICH patient groups with regard to their preoperative neurological status with quite different degrees of neurological impairment and not uniform consciousness levels, applying different surgical approaches and different intervals with regard to the onset of haemorrhagic stroke. Thus it is an essential issue to select appropriate patients and homogenous group for determining whether patients truly benefit from neurosurgical or not treated by stereotactic evacuation of the haematomas in the acute phase. In many recent studies the minimally-invasive method have shown to be highly efficient with little risk of re-bleeding and better short-term outcome [29-31]. Presently, some clinicians are exploring new methods to elevate curative effect of minimally-invasive operation technique. A study by Marquardt and coworkers focused on the use of a novel multiple target aspiration technique in 64 patients to aspirate a "sufficient proportion" of the hematoma with minimal risk for the patient. More than 80% of the hematoma volume was successfully aspirated in 73.4% of the patients with only one episode of re-bleeding [32]. Jose's study showed that CT-guided thrombolysis and aspiration was safe and effective in the reduction of ICH volume. But meantime they proposed further studies were needed to assess optimal thrombolytic dosage and must include controlled comparisons of mortality, disability outcome time until convalescence, and cost of care in treated and untreated patients [33].

MISPT is a new and novel operative technique obviously differrent from other various kinds minimally-invasive operations in design principle, which is developed by Pro Jia from China in 1994 with a distinctive thrombolysis installation, and highly safe and efficient functions of dissolving and draining coagulated blood. This treatment had been widely applied in China. According to some study in the past, it was presented that MISPT in acute ICH could efficiently clear hematoma, relieve hydrocephalus, drop the intracranial hypertension, and relieve the cytotoxicity of blood thrombin. Furthermore, the washing liquor could decrease the cytotoxic substances. In subacute hemorrhage, MISPT could reduce the neurotoxicity of the hemoglobin and its disaggregation such as ferri ion. This technique is characterized by its simple operation, not limited by equipment. The puncture of MISPT is little harmful for the brain, and profit the recovery of cerebral function, and the liquefaction technique contribute to the blood coagulum liquefied, which all help shorten the course of disease. In the whole procedure of the operation, patients were only treated with the puncture of 3 mm needle in diameter. Because of no gap between the needle and skull, which reduce the incidence of infection. Furthermore, it is not needed to open the skull and anesthetize generally, and being cheaper than other operations. Chinese National Research and Extension Community of the Minimally Invasive Operation suggested that MISPT is suitable to the cases that have hemorrhage volume > 30 mL in basal ganglia, and further standardize the operation indication, operation procedure and the applying methods of the hematoma liquefacient according to the random sampling of The Ministry of Public Health [34].

Although there being many studies to investigate the minimally invasive operation indication of ICH, few specially concerned indicatio of the MISPT. Furthermore, most of these investigations didn't establish the control groups strictly with uniform baseline characteristics patients on preoperation, analyze the correlation factors such as GCS score, hematoma volume, haematoma location, duration of BP and observe the long-term outcome for survival. In our study, above shortcomings in research were overcame with many factors being considered including preoperative neurological status, surgical approaches and opportunity applied, GCS score, incidence of complication and rebleeding incidence after surgery, long-term outcome 1 year after onset and so on. The result showed that the level of consciousness and GCS in MISPT were better than that of the CC group. There were 17 cases died in 20 patients with 4-5 GCS score in two groups(the ratio is 85.0%), the 3 survivor all treated with MISPT (table 1, one case referring to figure 2). The incidence of complications such as pulmonary infection, hemorrhage of digestive tract, and epilepsy in MISPT was obviously reduced compare to CC group. There were no cases of intracranial infection both in MISPT and the craniotomy group. The statistical analysis did not show the significant difference on the rebleeding incidence in the two groups(10.0% and 15.4% respectively, P = 0.151). There were no obviously difference between the case fatalities of MISPT group and CC group(18.9% and 24.4% respectively, P = 0.096). The long-term outcome of MISPT group surpassed over that of CC group according to the results of GOS, mRS and BI. (GOS, P = 0.000; mRS, P = 0.001; BI, P = 0.000).

In present, there were some exploration on thrombolysis methods. We used UK as thrombolysis methods in our MISPT study. Otherwise, in another cohort of ICH patients treated using FAST, volumetric analysis of ICH and perihematomal edema seems to suggest that local use of rtPA as thrombolysis which differed from UK used in our MISPT does not exacerbate brain edema formation. Furthermore, there seems to be a strong association between reduction in ICH volume and reduction in edema volume, as would be expected following the concept of ''hemotoxicity'' postulated by some investigators [35].

These above results indicated that the ICH patient meeting MISPT operation indication was unsuitable to choose CC. However, the patient with huge hemorrhage volume, or the state of illness progress rapidly, or in the early state of cerebral hernia should select the craniotomy to reduce the cerebral pressure. By the way, Some study have point out that although MIS on the patients with cerebral hernia may not get good curative effect, which can decrease the hematoma volume partly and reduce the intracranial pressure rapidly, and gain time for craniotomy [33]. Of course, CC has sometimes its superiority in treating ICH with bulk volume. Murthy combined the reduction pressure of removal bone flag and the clearance of hematoma to cure 12 patients with hemorrhage volume > 60 ml in the right hemisphere. As a result, 11 survivors were out of the hospital (92% of the survival rate), 6 cases of the survivors recovered well [36].

In conclusion, although larger randomized trials proving more relative benefits of MISPT treatment over traditional craniotomy or purely medical management are in process yet. However, our study and some other treatment trials about this treatment modality are rising with optimistic results superior over CC or PMT. Several methodological issues surrounding this form of treatment remain to be resolved, including comparison of the relative efficacies of various mechanisms of clot thrombolysis and drainage. If successful, MISPT for ICH perhaps becomes an important tool in the growing treatment armamentarium for ICH and with the potential for a disease modifying impact on these ICH patients. It is our hope that this ongoing study will bring us closer to other randomized testings regarding to more relevant factors with MISPT as an alternative treatment for ICH. Objectively, this MISPT are limited in their ability to achieve hemostasis and completely evacuate the hematoma. Proper selection of the ICH patient group in which to apply these therapies may hold the key to these advances.

## Conclusions

These data suggested that MISPT displayed its advantage in minute trauma and safety, and was feasible and to had a trend towards improved long-term outcome compared with conventional craniotomy.

## List of abbreviations used

ICH: intracerebral hemorrhage; MISPT: minimally invasive stereotactic puncture therapy; CC: Conventional craniotomy; GCS: Glasgow Coma Scale; PC: postoperative complications; RI: rebleeding incidence; GOS: Glasgow Outcome Scale; BI: Barthel Index; MRS: modified Rankin Scaleand; CF: case fatality

## Competing interests

The authors declare that they have no competing interests.

## Authors' contributions

ZHG, ZY and DQ conceived of the study, drew up the study's design and coordination, performed the statistical analysis and drafted the manuscript. LL, HW and XJZ conducted the minimally invasive stereotactic puncture therapy and conventional craniotomy in clinical work. HX and TYP helped to draft the manuscript. TYH participated in the study's design and statistical analysis. All authors read and approved the final manuscript.

## Pre-publication history

The pre-publication history for this paper can be accessed here:

http://www.biomedcentral.com/1471-2377/11/76/prepub
